# ‘Five minutes earlier, you were giving hope’: Reflections from interviews with doctors conducting assessments for South Africa’s childhood disability Care Dependency Grant

**DOI:** 10.12688/wellcomeopenres.18424.1

**Published:** 2022-11-25

**Authors:** Zara Trafford, Leslie Swartz

**Affiliations:** Psychology Department, Stellenbosch University, Stellenbosch, Western Cape, 7600, South Africa

**Keywords:** care dependency grant, social assistance, children with disabilities, disability assessment, decision-making, paediatric doctors, South Africa

## Abstract

**Background:**

In South Africa, medical doctors functionally decide whether a primary caregiver can access state-funded social assistance cash transfers (‘social grants’) for the care of their disabled child. In this paper, we unpack the subjective experiences of one group of doctors in the country’s Western Cape province who are involved in conducting assessments for the care dependency grant, designed to support the cost of disabled children’s specific needs.

**Methods:**

Individual in-depth interviews were conducted with five paediatric doctors who practice at three Cape Town public sector hospitals and regularly conduct assessments for the care dependency grant. Analysis was thematic and used deductive coding.

**Results:**

The doctors we interviewed were aware that care dependency grants were probably shared among household members but felt this was acceptable, as it contributed to the child’s wellbeing. Doctors seemed to be applying nuanced, individualised assessments but often felt the need to simplify the documentation of these assessments, sometimes even bending the rules, to ensure access. Doctors’ primary allegiance in these processes appeared to be to their patients. They identified more with their care responsibilities than their bureaucratic gate-keeping role but nonetheless felt the weight of their responsibility for decision-making, in the context of extremely strained public resources and a lack of guidance from the government’s social assistance implementation agency.

**Conclusions:**

The hyperlocal practices and approaches that doctors described allows for perception of the messier – but also more accurate – details of the system that is actually in place. Doctors’ narratives also reflect long-standing inattention to the ‘trickle down’ of guidelines to frontline implementers of disability-related grants. This cadre is a valuable but under-utilised source of evidence and information about the real-world functioning of disability-related grants administration and they should be actively included in implementation planning.

## Introduction

For most South Africans, the only access to income support for social protection is through a national, non-contributory social assistance programme. Various ‘vulnerable’ groups are eligible for poverty-alleviation or age- and disability-related unconditional cash transfers, known locally as ‘social grants’. The grants system in South Africa (SA) is relatively progressive, well-established and impactful (Fragoso, 2022; Granlund & Hochfeld, 2020; Kidd *et al*., 2018; Satumba *et al*., 2017). However, it is also large and costly, raising the associated economic, political and emotional stakes. Studies about the grant system, from the perspective of its recipients especially, report widespread implementation issues relating to systems for its administration, consistency, monitoring and distribution (Du Toit & Lues, 2021; Granlund, 2022; Hajdu *et al*., 2020; Hanass-Hancock & McKenzie, 2017; Kelly, 2013; Lund *et al*., 2009; Patel *et al*., 2019). As a result, questions of appropriate expenditure, the ‘deservingness’ of recipients and whether or not grants are getting to those who need them most are often central to investigations of the grants programme.

This is especially true for grants associated with a need for care due to age or impairment, as these are of a significantly higher value than poverty-alleviation grants. The ‘disability-related grants’ portfolio includes the care dependency grant (CDG), for the support of disabled children up to the age of 18, and the adult disability grant (DG), intended as income replacement for adults with long-term impairments or disabilities, as well as the small supplementary Grant-in-Aid (GIA).

There is a rich literature on the adult DG, partly because it has a large beneficiary base and is thus a productive site for investigation of state expenditure, the politics of redistribution and opportunities for reform or refinement of the existing system. The DG is also closely associated with perceptions of and actual issues relating to fraudulent activity in the grants system. This has prompted compelling discussions about the neoliberal undertones of state-funded social assistance, moral judgments about adults’ ‘willingness’ to work or ‘compliance’ with recommended action or treatment, and implications for citizenship and belonging in society (cf. for example Kelly, 2016a; Knight *et al*., 2013; Schneider *et al*., 2011a; Schnitzler, 2020). In other words, discussions about the DG tend to focus on adult labour market participation. Similarly, an extensive literature has thoroughly investigated the child support grant (CSG), the CDG’s counterpart for the support of non-disabled children, exploring a wide range of issues from the CSG’s impact on redistribution to its affective dimensions for different role-players, in various parts of the country (cf. for example Gordon *et al*., 2017; Granlund & Hochfeld, 2020; Hajdu *et al*., 2020; Patel & Ross, 2022; Zembe-Mkabile *et al*., 2015). In conversations about social assistance for disability, then, the emphasis is on working-age adults. In conversations about social assistance for children, the emphasis is on non-disabled children. Even the Social Relief of Distress grant, instituted and extended during COVID-19, has received much recent attention due to its impact on adults without disabilities but under the eligible pension age (Gronbach *et al*., 2022; Köhler & Bhorat, 2021; Marx *et al*., 2022). In contrast, the CDG brings a rather different set of focal points into view: the human rights of disabled children, the need to support their caregivers in accessing insufficient and inaccessible public services and the question of income replacement for these caregivers, who are often forced to stay away from work because they must provide full-time home care for their child in the absence of alternate support systems.

The CDG has received minimal research attention to date, compared with other grant types. The small literature specifically investigating the CDG that is available is focused on caregiver experiences (Dimhairo, 2013; Letsie, 2016) and desk-based analyses of policy frameworks (Khumalo, 2020; Martin *et al*., 2014), as well as two now outdated quantitative and mixed-methods sociodemographic assessments (De Koker *et al*., 2006; Delany *et al.,* 2005). An additional range of research touches on the CDG and its recipients (cf. De Sas Kropiwnicki *et al*., 2014; Elphick *et al*., 2015; Hall, 2022; Philpott, 2014; Philpott, 2018; Saloojee *et al*., 2007) but this work has not explored the grant’s administration from the perspective of its implementers. No published research has investigated how health workers tasked with assessing eligibility for the CDG feel about the process, how they perceive potential applicants and actual recipients and how they balance their dual role as health workers and bureaucratic agents of the state. These questions are important because they help to illuminate the real-world implementation (Hoag, 2010; Hoag & Hull, 2017; Hull, 2012) of social assistance policy in SA. The question of how to decide upon an approach to disability assessments for state-subsidised benefits is not a uniquely South African problem (Bloch, 2007; Gerst & Schwitzman-Gerst, 2019; Johnson & Nettle, 2020; Palmer & Harley 2012). The implementation of strict cut-off thresholds for disability benefits will always be challenging, partly because disability manifests along a wide spectrum and in individually-specific ways. This fluidity resists simplified categorisations and sits uneasily with the harder lines usually required to make decisions about who can or cannot gain access to publicly funded benefits. However, this debate is thrown into stark relief in SA because of the extremely high levels of poverty locally, which intensify the challenge of balancing fairness and compassion because technically, much of the country’s population would benefit from income support. In comparison with some other low- and middle-income countries, SA’s profound income inequality also occurs within a context of relatively sophisticated infrastructure, resulting in chasmic gaps in quality of life, depending on whether or not an individual can pay to access services.

In this small study, we aimed to discern and reflect on how one group of specialist paediatric doctors who are based in hospitals in SA’s Western Cape province approached the CDG assessments they conducted regularly. We wanted to understand how doctors reacted to patients and their families in the process, how they understood CDG policy and what their interactions with the country’s social assistance implementation agency were like. In order to try to manage limitations in the public sector health system and the lack of additional interventions for state-subsidised social protection, these doctors have developed a range of practices around disability-related grants assessments along similar but also contrasting patterns to practices that were previously reported in a study of assessors for the adult DG (Kelly, 2016a; Kelly, 2016b; Kelly, 2017; Kelly, 2019). These bottom-up practices seem to have been influenced more by paediatric doctors’ own medical expertise and insights from colleagues than by any formal guidelines from the country’s official social assistance implementation agency, the South African Social Security Agency (SASSA), or its parent government department, which governs the social assistance legislation. These health workers’ ‘informal’ solutions to the problem of operating within a highly dysfunctional system represent a knowledge base that is often hidden because doctors do not necessarily have the time or space to reflect on their practices. Importantly, all of the doctors we interviewed were also ‘treating doctors’, meaning that they generally had some kind of relationship with the people they assessed for the grant, which is not the norm with disability-related grant assessments across the country where many assessing doctors may not know the applicant. Nonetheless, as we shall show, when these doctors working at the coalface of grants implementation have the opportunity to reflect with interlocutors on the complexities of their work at the interface between health care and the social development system, they provide interesting and important information. Gaining a better appreciation for the actual implementation of assessment processes – and then feeding these insights back into ongoing attempts to refine implementation – is also important because, as others have argued, the frontline practices of assessing doctors have much more of an influence on access to the CDG than the eligibility or access guidelines that may be dictated by official policy (Kelly, 2016a).

## Background: The grants system in South Africa

All grants in SA are legislated by the national Department of Social Development (DSD) and managed and administrated by SASSA, the DSD’s social assistance ‘implementation agency’. CDGs are currently disbursed to a primary caregiver a child with a long-term disability from the time of approval until the child turns 18. The value of the CDG was at ZAR1,980 (approx. USD112/GBP98) per month in September 2022 (SASSA, 2022), around four times the amount available to recipients of the CSG, which was ZAR480 in the same period. As of September 2022, there were 155,711 beneficiaries of the CDG nationally, accounting for 0.8% of the total grant recipients in the country, which is significantly lower than the amount of recipients of the adult DG and the CSG, accounting for 69.9% (1,044,084) and 5.5% (13,173,267) of the total recipients respectively (SASSA, 2022b). The distribution of social grants is governed by the Social Assistance Act (SAA) 13 of 2004 (amended in 2008, 2010 and 2020), which stipulates that access to the CDG is contingent on 1) a means test to determine the income of the applicant, and 2) a (medical) assessment of their child’s impairment (South African Parliament, 2004). However, the governing legislation contains no explicit instruction about where the threshold for CDG inclusion or exclusion lies. Rather, an eligible applicant is defined as the primary caregiver of ‘a child who, due to his or her physical or mental disability, requires and receives permanent care or support services’ (DSD, 2022). The 2004 Act’s original description of a ‘care-dependent child’ included reference to ‘severe physical or mental disability’ but the concept of severity was not included in the description of CDG eligibility. Later amendments to the Act were internally contradictory as they preserved the concept of severity in the Definitions section but once again, did not include severity as a metric in the description of CDG eligibility (DSD, 2022). However, SASSA’s website and public handout brochure currently indicate that only children with ‘severe mental or physical disability’ (emphasis added, SASSA, n.d.-b; SASSA, n.d.-a) are eligible, which is echoed on the national government website (South African Government, n.d.) and the Western Cape government website (Western Cape Government, n.d.). Based on existing legislation, it is unclear whether or not, or to what extent, severity ought to be included in assessments of eligibility. In fact, concurrent interviews with SASSA officials suggested that the extent of a child’s care needs should actually be an assessor’s primary consideration for approval or rejection of CDG applications (Trafford & Swartz, 2021).

Historically, medical doctors were the only cadre of health workers permitted to conduct disability-related grant assessments in SA. Following a series of attempts circa 2008–2012 to redesign and make these assessments less subjective, more comprehensive and inclusive of greater community impact (Kelly, 2016a; Schneider *et al*., 2011b) by using multi-person assessment panels, the system defaulted back to individual doctors being tasked with making these decisions, as is currently the case (Kelly, 2016a). This is reportedly especially true for the CDG in the Western Cape province, where the majority of CDG approval forms reportedly come directly from doctors based at secondary or tertiary hospitals, via their social work departments (Trafford & Swartz, 2021). In SA’s eight other provinces, doctors who may be separately working in the private or public sector are ‘directly contracted’ by SASSA and are paid per assessment. In the Western Cape, SASSA does directly contract a small group of doctors but most conduct assessments under the auspices of a service-level agreement between the provincial Department of Health (WCDoH) and SASSA’s regional office. Thus, most grant assessing doctors are actually employed by the WCDoH and generally work independently of SASSA. SASSA’s slogan is, ‘Paying the right social grant, to the right person, at the right time and place. *Njalo* (every time)!’ This reflects SASSA’s primary intention: that grants are awarded to ‘deserving’ applicants, i.e. to those who meet the relevant eligibility criteria for each grant. The converse implication of this is that those who are technically ineligible but gain access anyway are ‘unworthy’ recipients. However, although there is an official relationship between SASSA and the WCDoH in place, there is a conspicuous lack of guidance available from SASSA regarding the thresholds that affect how doctors perceive whether or not an applicant is eligible. This is partly because according to officials in regional and national leadership, SASSA officials characterise themselves as administrators and bureaucrats, not disability practitioners, so they do not feel equipped or appropriately positioned to guide doctors in their assessments of disability (Trafford & Swartz, 2021). Thus, the already difficult work of defining the eligibility of an applicant (Kidd *et al*., 2018; Moodley, 2021) functionally falls to individual doctors, who are not systematically provided with formal instructions about whether or not to include severity as a metric for access, nor what the threshold for severity should be.

## Methods

### Ethics

Ethical approval for this research with human participants was obtained through the Stellenbosch University Research Ethics Committee: Social and Behavioural Research (REC: SBER). Initial approvals were received on 28^th^ November 2019 (PSY-2019-13097) and renewed annually until data collection had concluded. Procedures were in accordance with the ethical standards of the REC: SBER, as well as the 1964 Helsinki Declaration and later amendments. All interviews were remote and additional ethical permissions, including relevant safety and data storage procedures, were gained prior to data collection. Written informed consent was obtained from each participant prior to interview. Data were stored on password-protected cloud storage and backed up weekly to an external hard drive. Only the first author has access to these data.

### Participant demographics and recruitment

Following a request for permission to conduct remote interviews with hospital-based doctors, provincial health research management informed the first author that no research would be approved during what was then the ‘third wave’ of COVID-19 infection surges in the province. In late November 2021, permissions were obtained from doctors’ line managers or heads of department, prior to initiating contact with participants. Recruitment for remote interviews was then conducted by the first author by means of direct email contact, using email addresses gathered from the websites of the health facilities where these doctors worked or the academic institutions where they had joint appointments. In one case, an earlier participant suggested additional possible participants. Suggested participants were then approached independently by the first author, without including the referring participant in the recruitment invitation. Potential participants were sent an email which summarised the goals of the research, as well as what would be expected from them in terms of time commitments and related arrangements. A one-page description of the overall research project was also included. The first author approached six potential participants in this way, and five agreed to be interviewed, while one did not respond.

The data from interviews with assessing doctors presented here were gathered as one section of a broader multi-stakeholder qualitative study to investigate the role of and perceptions about the CDG. In the original study design, the proposed sample of doctors was flexible but estimated at 5–10 participants. The decision was made to stop recruiting doctors after interviewing five participants, as data collection had to conclude by April 2022 due to funding and other timelines. The doctors interviewed for this study were all based at specialist hospitals in Cape Town, the capital city of the Western Cape province. All indicated that CDG assessment was a daily or very regular task for them. All participants were neurodevelopmental paediatricians with at least a decade and as many as thirty years of experience each. All were cisgender, with four being women and one a man. However, we have used she/her pronouns for all direct quotes, to maintain a degree of anonymity. None of the participants identified as disabled and their ages ranged from 39 to 65 years old. Most had joint academic appointments in parallel with their clinical roles. Participant identifiers (Dr A, Dr B etc.) have been randomly assigned.

### Data collection and analysis

Data were collected from June 2021 to May 2022 using semi-structured individual in-depth interviews (IDIs) of about 1.5 hours long. The first author conducted these remotely (due to COVID19-related prohibition of in-person research) and used videoconferencing software of the participant’s choice. Two participants selected Zoom and the others were interviewed using Microsoft Teams. All were also recorded using a Zoom H1n audio-recorder. The first author transcribed all recorded audio data verbatim, a process which served as initial data familiarisation. Transcripts were then reviewed multiple times for additional familiarity. For this manuscript, deductive codes were used to highlight common or unusual responses to our semi-structured interview questions. An initial version of this manuscript that comprised the results of a basic thematic analysis was drafted by the first author and shared with the second author, who suggested a narrower focus on the subjective experiences and practices of the doctors included. All findings were discussed with the second author, who was also involved in conceptualisation, edits to and approval of all drafts of this manuscript.

### Trustworthiness

While these are not quoted in this article, interviews with other role-players (caregivers, SASSA staff, civil society representatives and key informants) were also included in the parent study, allowing for comparative reflection. We have also included detailed descriptions of our setting and the administrative arrangements at play in our participants’ context, to allow for some degree of transferability (Korstjens & Moser, 2018). By including other reflexive considerations throughout the Results and Discussion, we have tried to make explicit the ways in which our interpretations or participant narratives may be affected by bias or lack of direct observation. However, this work was intended to be exploratory, rather than to aim for generalisability or widespread transferability. Our participants’ insights offer directions for future research, aim to create more conversation in this under-researched area and add to the growing international understanding that frontline worker perspectives are critical for gaining a deeper understanding of the real-world functioning of complicated bureaucratic systems.

## Results

### Doctors’ feelings about CDG applicants

Doctors framed the CDG as useful for improving a family’s access to the means for early and ongoing intervention and support for their disabled child. This was because they felt that having an income would ‘get this mother or parents empowered… to access early childhood development services’ (Dr E). However, they also explained that in light of insufficient state support and service delivery, the grant was ‘not enough’ for the majority of caregivers, especially those who were very poor or had a child with multiple specific needs. Sometimes, this meant that when a child was assessed they might technically not qualify for the CDG but from the doctor’s perspective, their disability would inevitably develop into something more ‘severe’. These doctors then approved the CDG, in anticipation of the child’s long-term prognosis and reasoned that the child would probably need this support at some point anyway, so they ‘would rather err on [the side of] offering [the CDG], even though you’re not sure’ (Dr E).

Some participants said that their own emotional reactions to the desperate circumstances of patients and their families could make it more likely that they would approve the grant because ‘we all pander to the sympathy effect, where you can see a parent really struggling’ (Dr E). This was sometimes the case even when a doctor believed that the child’s disability might not strictly qualify for the CDG.

‘…there have been times when my judgment has been clouded by the way a person presents… when you get your heartstrings pulled… [and they say], “life is really hard” – I’ll [think], “Ugh, grant [approved]”‘ (Dr D)

Having acknowledged the powerful emotional reactions she had to the difficult and economically perilous lives of her patients, Dr D also noted that she sometimes had negative reactions to potential applicants. This was particularly the case if, for example, caregivers had missed many months of appointments for their child and then arrived and asked the doctor to fill in a grant form.

‘…there are also people that I have rejected because I have a sense that there’s a very strong pecuniary interest in why [they’re] coming to see [me], and the child is secondary… I want to think that I’ve never gone, “You’re bad, you can’t get it”. But it’s just that, for many, many grant applications, you just have to close your eyes and hope this isn’t going to meth[amphetamine]… and that someone’s going to care for the child’ (Dr D)

However, as in the case of this example she provided, this doctor’s negative reactions were usually based on her awareness of wider social problems and their effects on families, rather than judgement of the person directly in front of her. Doctors who participated in this study tended to opt for more rather than less inclusion, even if this meant they sometimes had to, as Dr D put it, ‘close your eyes and hope [for the best]’.

Doctor participants expressed deep empathy for and sensitivity to the circumstances facing potential CDG applicants. Although uncertain about exactly how CDG funds were spent, doctors felt that the CDG was probably often subsumed into the household income. One doctor noted that for poor parents, it was ‘very difficult to just keep [CDG funds] for one child when you have another child next to you who’s hungry’ (Dr C). Although this meant that the CDG was not necessarily used for the disabled child alone, Dr C went on to explain that ‘if the rest of the family is not ok, there’s no way that child is going to be ok’. As such, the use of these funds for the household was not framed as problematic and participating doctors drew a direct link between the stability of the household and the quality of care available to the child with specific needs. They felt that if caregivers were able to cover the costs of the household, the ‘wellbeing of the entire family and mostly, of the child who’s most vulnerable, [would be] improved’ (Dr E).

### Severity thresholds: a moving target?

The subjectivity of severity thresholds was a prominent thread in conversations with doctors who participated in these interviews. An experienced doctor who had been in practice since the late 1980s also reflected on her shifting perceptions and depth of understanding of the need to consider each disabled child individually and the uncertainty surrounding severity. She noted that when she had first started doing assessments, she had rejected CDG applicants if ‘the[ir] child was continent, could attend a mainstream creche, [or] was ambulant’ (Dr B). In other words, she had only approved the CDG if a child’s disability was so severe that they could not perform any functions of daily living. However, around 2008 Dr B had started to soften her approach as she realised that ‘you’ve got to see each case individually – just because a child can walk, doesn’t mean they don’t need a disability grant!’ The usually less visible demands of caring for neurodiverse children, in particular, were also highlighted by multiple doctors.

The other participating doctors shared similar sentiments and felt that as well as being more clearly defined, the severity threshold they perceived as being in place ‘could be lower’ (Dr A). They indicated that even children with moderate disability can have very high care needs and worried that enforcing very strict guidelines could have negative implications for the future prospects of a large proportion of children who might not be ‘severely’ disabled, but were nonetheless in desperate need and might actually benefit most from the therapeutic interventions that the CDG could facilitate.

‘…take the example of a child who’s got a very mild hemiplegia… [a] weakness on [the left] side of the body. They can walk… [their] language is normal. But… they’re clumsier, they can’t run as well, can’t manage stairs… And maybe they have some significant learning difficulties… In a [mainstream] class of 45 or 50 children, their difficulties are not going to paid attention to… The hand, which with the right kind of intervention could become at least moderately functional, actually has no chance… [but] they wouldn’t be eligible for a [CDG]… [That] gives you an idea of how the milder children can end up. They’re the ones that in a way, have the best potential of being able to [live] a relatively optimised, maximum potential life… but ironically, because they’re “too” mild, they don’t get anything, and so you get the worst outcome for them and we [end up only] supporting the very severest end of the spectrum’ (Dr A)

Despite the lack of official guidance from SASSA, participating doctors found most CDG judgments reasonably straightforward. However, all of them also indicated that there were always ‘grey areas’, which then required them to make personal judgments on the threshold for severity because of the lack of ‘clarity of definitions about what “severe” means’ (Dr A). The same doctor commented that ‘on [disability-related assessment] forms, it’s a zero-sum game [but] in reality, there’s a lot of grey’ (Dr A). Another noted that the process was often ‘very subjective, especially when it comes to moderate [disability]’ (Dr C).

In order to secure access for patients they had decided were deserving, doctors reported that they were sometimes compelled to be dishonest about the severity of a child’s impairment because of their belief that children with moderate disability would not be approved for the CDG. Based on prior experiences with clients who in their view ought to have been approved but who had had their applications rejected by SASSA, doctors felt the need to be very explicit or sometimes even to exaggerate. This was also because of the use of a specific form, as detailed further in the following section.

‘There is a big group of kids who may be moderate but… should get the CDG… [but] on the form it’s [only] “mild” [or] “severe”… I can honestly say I end up choosing severe just so they get that grant… [When] I was in general paediatrics I would write moderate, [but] the feedback was that they never got [the grant]’ (Dr C)

Doctors worried that a more accurate, nuanced approach to documenting their assessments of children’s impairments would not secure the grant for these applicants.

In short, once these doctors had made their own assessment during their in-depth treating sessions with their patients, and had decided the potential applicant was deserving of support, they aimed to do what they could to ensure approval. A few doctors commented that contemporary understandings of disability (specifically neurodiversity) were not reflected in the systems and tools they used, which could again lead assessing doctors to bend the rules:

‘…a lot of my autistic [patients] will probably be able to do some of the mental tasks [on the form]… [so] now what do I do?! Because they’ve completed the test, but they can’t even have a conversation… then it gets down to, is this child really difficult to live with, and will they be able to get on a bus and go to school? Never! But I don’t have a tick box for that one. So I complete the form with an appalling precis of the child, for SASSA’s purposes… I have to have this absolute black paint, over the child’s assessment, to ensure that they’ll get the grant’ (original emphasis, Dr D)

These doctors saw this rule-bending as a necessary means to a positive end.

However, doctors *were* worried that caregivers would read and be discouraged by assessments that so strongly emphasised the severity of their child’s impairment, even though ‘five minutes earlier, you were… giving hope to that same mom, about their child who’s doing so well’ (Dr E). This conflicted with doctors’ medical and therapeutic intentions, which were reliant on the caregiver feeling hopeful about their child’s prospects.

‘I know that I’m not jacking the system, I’m just making sure that [they get the CDG for their child who can do some things but]… is [still] incredibly dependent… [So] I say [to parents], “Please don’t read this form, it’s not the truth about your child – I have to say these things, because if I don’t… you’re not going to get a grant”‘ (Dr D)

In these instances, doctors aimed to communicate clearly with caregivers, to help them understand that these occasional exaggerations or overly simplified descriptions of their child’s impairments were there to secure their CDG access and ‘to explain who is processing the form… [and] that they’re not medically trained, so I need to spell it out in simple words’ (Dr E).

Doctors were required to serve, in these moments, at a difficult intersection between the emotionally weighty task of bolstering the hope of these parents while initiating them onto a grant that could be perceived as a marker of their child’s ‘hopeless’ future. It seemed much more important to these doctors that they deliver on their clinical role than that they follow (their perception of) SASSA’s rules to the letter. One noted that ‘experience… and being around the block as long as we have’ had taught them ‘which diagnoses fulfil [SASSA’s CDG] criteria’ (Dr E). In these instances, doctors seemed to be trying to achieve two goals at the same time: addressing their bureaucratic partners (SASSA) in terms that would be understood, and pre-empting or preventing additional expenses and rejection for their patients. Rather than being unsure about how to gauge the impact or medical severity of the condition of the children they treated, they were aware that these assessments would be received by SASSA and aimed to ensure that when they believed an applicant was eligible, the applicant’s access to the grant would be relatively straightforward.

### The ‘white form’: reflecting on hyperlocal tools and processes

Participant doctors reported that SASSA and the DSD did not provide explicit inclusion and exclusion criteria, and nor did they provide specific information about how to conduct assessments. Instead, participating doctors had usually relied on a line manager or senior colleague within the health facility where they had first done these assessments for guidance in gauging qualification for the CDG. Despite conducting CDG assessments since 1997, for example, Dr D commented that she had ‘never encountered anything official’ in terms of eligibility guidance. All of these doctors also had joint academic appointments, so they supervised medical students and taught them ‘how [the CDG] fits into care pathways and the management plan for children who fall under our care’ (Dr A). Tools and practices sometimes travelled from one hospital to another, but this appears to have happened informally, without an underlying structure or organising logic. As a result, institutionalised patterns regarding tools and procedures have sprung up, especially in relation to the CDG, with assessing doctors technically serving as key frontline implementation agents of social assistance for disabled children and their families.

One example of how such hyperlocal, institution-specific practices have emerged, multiplied and carried over from individual doctors (and their students) to others, is the use of the so-called ‘white form’. Official barcoded SASSA forms for disability-related grant assessment, rolled out around 2008, were not being used in the settings where participant doctors worked. Instead, they used the white form, an older version of the CDG assessment that had been used in their hospitals for many years. Some participants had ‘never seen’ an alternative form but even when doctors were aware of the official form, they preferred the ‘white form’, with one doctor succinctly explaining why this was the case: ‘all I’d really like to do, at the end of my work, is just press the green button – failing that, the ability to quickly complete a form is a godsend’ (Dr D). Having seen some of the newer forms, Dr A explained that they were ‘much more woolly (unclear) with respect to what a child can and can’t do… I think [because] it’s been designed by somebody who isn’t necessarily medical?’. Dr E indicated that after moving from a tertiary hospital to a different hospital, she had ‘told SASSA that I would be using the same form I use at [my other hospital]’ and they had accepted this. With a wider view of the shortages in the system and an awareness of how few doctors are willing to conduct grant assessments, it may also be the case that SASSA is trying to make things as easy as possible for these doctors, in order to ensure that they keep participating in assessments and are retained as a resource in the grants system.

Although doctors who had also seen or used the newer SASSA form opted for the white form, they nonetheless considered it ‘far from ideal’ (Dr E). In cases of ‘obviously severe’ (Dr C) disability, the white form’s questions felt unnecessary. Simultaneously, the form could not capture the nuances of impairments that were medically moderate but might still be having a considerable effect on the child’s independence and on the scale of care and attention required from their caregiver. The white form was favoured only because it was efficient and reasonably well-aligned with their existing diagnostic approach, as captured in the following reflection:

‘I use it because I can do it in my sleep. Our speciality is very paper-labour intensive… [but] you can still use the white form as part of your normal consultation… [so] it’s user-friendly [because] you can just scratch [irrelevant questions] out… [I also use it] for uniformity, because state patients largely get their [CDGs] from [specialist hospitals], which use the white form’ (Dr E)

Ultimately, doctors used the white form because it allowed them to achieve more quickly what they felt was their most important function: ensuring that their patients obtained social assistance so that they could best support their disabled child’s needs.

Doctors seemed able to assert themselves with SASSA and SASSA, in turn, appeared to be lenient with doctors’ use of the form they preferred. One of the implications of this looseness and flexibility, however, was a profound lack of support. In order to manage their hefty task, doctors were effectively taking advantage of the gaps and loopholes they knew existed in the system to obtain support for needy families. Knowing that there were huge problems with the overall functioning of the system, they operated as well as they could within these constraints, used the forms that were most efficient for them (and their patients) and applied thresholds on the basis of their own historical and practice-based knowledge, rather than a clear guideline from the entities actually responsible for administering and managing the grants system. Doctors were forced into developing their own mechanisms for balancing the demands of their clinical work with the need to connect patients to income support, a key pathway to care. In the absence of proper guidance, the doctors we interviewed seem to have established various pragmatic approaches that worked well for them in the context of their day-to-day work, while also meeting (their perceptions of) SASSA’s requirements and assisting desperate families in accessing financial support.

### Between a rock and a hard place

Participating doctors unabashedly appreciated the flexibility that the white form gave them. However, they did express conflicted feelings about the wider societal implications for fairness, consistency and state expenditure. For example, one doctor was insistent that although it was difficult to see patients in desperate circumstances, she would never have approved the grant on the basis of poverty alone because in this role, doctors ‘are responsible [not just to their patients but also] to the state and to the government, [so they] have to fill [grant assessment forms] in with integrity’ (Dr B). In personal communications subsequent to our interview, Dr D noted that she would value seeing ‘an expanded vocabulary or debate on what constitutes “enough” dependence to warrant a [CDG] in this country in the 2020s’. This doctor was drawing attention to the fact that because most patients accessing the public health system were living under difficult circumstances without enough support, it was sometimes hard to know who should be included or excluded. The same doctor added that although this debate might be happening in policy or legislative contexts, ‘the trickle down [to the frontline] hasn’t happened… in the real world of my clinic, I just don’t see [policy] driving practice’.

To varying degrees, doctors were concerned about consistency within their own and across other doctors’ judgments, mostly because of the known weaknesses in monitoring and uniformity across the disability-related grants assessment system. In our interview, Dr D had eloquently and movingly noted the weight of responsibility for ensuring fairness in the social assistance system, as well as her concerns about the public fiscus.

‘It’s a really hard space to be in… Sometimes I’ve just kind of prayed for some protection there. And most of us, because we’re softies, will just kind of [approve the grant] but you can see how that plays out in millions and billions… When I started training, there was always a sense that there was a SASSA doctor who vetted the forms… [so] that we weren’t the final arbiter of whether a child got a grant or not. It was always a recommendation. But I have not had any contact with anything resembling a SASSA doctor who’s actually pushed back against an application… It does worry me that there is a massive bill… But I don’t see anyone pushing back at me, even though I’ve written my telephone number there for twenty years! … I would really appreciate that, just once, to tell me that someone out there is listening and watching a system that must cost the state… a lot of money… This morning I had a phone call with a parent whose child… [has] Down syndrome [with] moderate intellectual disability… his heart is fine and he’s just [moving] around like any old five-year-old, with clear intellectual disability, but not of a type that warrants a family to suspend life and do inordinate amounts of caring. [Technically,] I don’t think that child should get a grant, but I know there will [then] be an internal consistency within [my assessment history], because I’ve probably [recommended] grants for 95% of children with Down syndrome… that oversight [from SASSA]… is missing’ (Dr D)

This participant (and others) actually seemed to be craving more and much clearer guidance from SASSA, so that they could worry less about fairness in each assessment and could also share the responsibility for this enormous task. They were happy to advise on impairments and their impact, as this was a task they felt equipped for and agreed was not SASSA’s speciality, but it certainly felt wrong to them that it was their responsibility alone. Without proper support from the state’s social assistance implementation agency, doctors were forced to try and balance giving people help while being aware that resources were extremely strained, which Dr A described as ‘a wicked problem’.

When asked how they thought the system’s limitations might be addressed or improved, participating doctors expressed a willingness to adjust their own behaviours but did not have much hope that the country’s ‘thin administrative capacities’ (Dr D) and lack of human resources for health offered much scope for improvement. Doctors also worried about the perceived lack of commitment at SASSA, as reported by their patients. They especially noted that officials tended to be ‘anonymous’ (in contrast with named, treating doctors who regularly had contact with their patients), leading to poor accountability and transparency.

‘SASSA… [is] a big, grey hole, isn’t it? We don’t know who we’re dealing with. We hear anecdotal stories – true of most government departments in this country – of people who are rude, and people who are inaccessible, and people who are incompetent… [it’s] not a user-friendly service for already strained parents in this country who… [have] a child who has hugely specialised needs and they are struggling financially, emotionally… having to get into this massive… queue, only to get to the front to be chased away because you didn’t bring this and you don’t know that. It’s not streamlined, there’s no open lines of communication’ (Dr E)

The doctors we interviewed all also worried about a single, cash-based intervention being used in place of more substantial or complementary interventions for the social protection of disabled children and their families, such as educational subsidies for children who might be able to access mainstream schooling with appropriate support.

The connection between SASSA and CDG-assessing doctors employed by the WCDoH is clearly mostly logistical, rather than being based on shared goals or understandings. This may differ somewhat in the country’s other provinces, where directly contracted doctors are the majority of the assessing workforce and might be provided with more formal ‘nudges’ by means of the official SASSA form. But in the Western Cape, no formal guidelines or training are systematically provided to these assessing doctors. Investigations of bottom-up practices, such as this study, capture some of the realities ‘on the ground’ and show how critical concepts (such as ‘severity’), which are at the heart of this conversation about public benefits, are not agreed upon or understood in the same ways by the key role-players involved.

## Discussion

Besides one notable study that specifically focused on the experiences and opinions of adult DG assessors in the Western Cape (Kelly, 2016a; Kelly, 2016b), there has been little insight into the perspectives of doctors who conduct disability-related grants assessments in SA. Specifically, no previous research reports on doctors’ narratives about conducting CDG assessments. The Cape Town paediatric doctors we interviewed seemed to be applying a nuanced and highly individualised approach to CDG assessments. They were keenly aware that while (medical) severity can correlate with higher care and support needs, this was not necessarily the case for all disabled children. They were also aware that, as is the case with most grants in the country (Gutura & Tanga, 2017), the CDG was probably distributed among household members rather than being used exclusively for disability-specific needs. Doctors felt that this was an appropriate use of the grant, as improving the overall wellbeing of a family would help their disabled child by extension. Further, participating doctors explained that children with conditions that were medically moderate might nonetheless have substantial therapeutic or other support needs. Doctors knew that if these children did not gain access to the larger CDG, their families would likely only be able to find similar support through the much smaller CSG. Caregivers would then be less able to present at hospital for their child’s medical care, to pursue other therapies or assistive devices or to pay for accessible transport or specific nutritional support, with negative implications for their disabled child’s health and happiness.

Doctors’ narratives about feeling obliged to bend (what they perceived as) the rules shows how, in the moment of assessment, they had to try to make complicated calculations about need, deservingness and the unlikely chance that these children and their families would find sufficient support elsewhere. Some doctors worried that because of their perception that severity was the primary condition for access, the CDG might only be functioning as a survival mechanism for its recipients, rather than as a means for genuine social protection. Doctors also knew that SASSA could desk-reject disability-related grant applications if the child’s impairments were described in accurate terms that another disability practitioner would understand, but which could not be reduced to one of three possible boxes to tick. As such, as in the case of some of the hospital-based adult DG-assessing doctors from previous research, this had involved ‘selectively applying and ignoring guidelines as it made practical sense’ (Kelly, 2016a). Paediatric doctors in our study seemed to be simplifying their CDG assessments because they understood that there were no health practitioners checking the forms and did not feel that non-clinical administrators were properly equipped to understand nuanced assessments. Individual doctors bore the weight of this decision-making but also felt unable to be more accurate in their assessments, because of the spectre of SASSA’s potential rejection. Indeed, some doctors noted that although they would not have been satisfied applying assessment processes that did not incorporate in-depth knowledge of child disability, they also did not want to have to be solely responsible for the fair and equitable distribution of needs-based grants. In these instances, they were caught between compassion for their patients’ circumstances and a wider commitment to fair, distributive justice.

In contrast with the aforementioned study with doctors who worked in the same province and conducted mostly adult DG assessments, the doctors we interviewed did not dwell on the ‘worthiness’ of potential CDG applicants during our interviews. They *were* concerned about those most in need gaining access but also said that they would prefer to make inclusion errors than to exclude potentially deserving children. They shared concerns about the distribution of limited state resources, but this was not their primary focus. Instead, doctors were profoundly aware of the fact that the CDG was the only predictable, steady source of support from the government for these families and their disabled children and seemed to lean toward lenience, rather than being especially focused on whether or not the applicant was, strictly speaking, ‘deserving’. Kelly (2016a; 2016b) theorised that her doctor participants, who mostly conducted adult DG assessments, were affected by a range of factors including their environment, relationship with SASSA and wider patterns including the rapid growth in DG beneficiary numbers when treatment for HIV had not been available in SA, the high incidence of fraud and ‘malingering’ associated with the DG and experiences that doctors had had with threatening applicants. Many of the doctors Kelly interviewed had adopted an apparently quite stringent approach to grants assessment, erring more on the side of exclusion when they were uncertain. In these cases, doctors defined worthy applicants as those who were deserving of support from the government’s public fiscus because they precisely met SASSA’s eligibility requirements. This was not without exception; some of the doctors that Kelly worked with were more lenient, emphasising the importance of empathy in considering their patient’s socioeconomic circumstances. These ‘two extremes in the way that doctors frame[d] and deal[t] with DG assessment’ were described by one of Kelly’s participants as ‘hard’ and ‘soft’ doctors, respectively (Kelly, 2016a). Kelly further theorised that doctors’ judgements of grant applicants seemed to have been motivated by overlapping ‘framings’ that doctors consciously or unconsciously applied to DG applicants, based on the doctors’ own personal characteristics and experiences such as age, training, social context, belief system, political leaning and so on. The doctors we interviewed for this study seemed to tend toward the ‘softer’ approach, with one even contending that ‘we [i.e. paediatric doctors working in specialist hospitals] are softies’. They were sometimes conflicted about this, however, and the same doctor noted specific fears about neglectful caregivers’ potential expedience, which could lead to rejection, and the possibility that grant money could be used for drugs. However, she still commented that she was usually inclined to approve access to the CDG, in the hope that at least some of these funds would get to the child with specific needs and contribute to their improved quality of life.

SASSA has expressed concerns about the bias and subjectivity of doctors, framing this as an impediment to standardisation and fairness across the disability-related grants portfolio (Kelly, 2016a; Kelly, 2017; Mitra, 2010). In addition, SASSA officials in the Western Cape and at national level are clear that although they tell clients that SASSA is deciding whether the grant will be awarded, the decision is actually being made by doctors, whose CDG recommendations are generally accepted by SASSA (Trafford & Swartz, 2021). While we can only theorise the reasons for the relative lenience of doctors in this study, it may be partly due to there being much less public concern about CDGs being fraudulently accessed and the existing beneficiary numbers being relatively low. In addition, in contrast with the DG, which is very well-known in the general population and is regularly requested by hopeful but ineligible applicants, CDG assessments were generally initiated by doctors and patients did not often request the CDG. Finally, a key difference between prior research with assessors and this study was that in the former instance, some of the doctors conducting assessments for adult disability grants were contracted by SASSA and were not necessarily providing treatment at the same time (Kelly, 2016a). For those who were also treating the patient they assessed, the context was less objective but ‘treating doctors were more likely to feel a sense of responsibility towards their own patients’ (Kelly, 2016a). Assessing doctors who also treat potential CDG applicants are likely to have direct and ongoing care relationships with many of these patients, which may affect how doctors respond to being forced into the position of judge and jury. Similarly, our participants often bundled their CDG assessments into a consultation or diagnosis session, which may, for them, have foregrounded their clinical role and increased their sense of commitment to care. They may also have been more inclined to approve a CDG, in comparison with an adult DG, because the individual at the heart of the CDG is a child and children are usually reliant on an adult and have less social autonomy, suggesting that they are less likely to ‘malinger’ or try to gain access fraudulently. This may have softened their reactions. Whatever their reasons, the doctors who participated in our study seemed most attached to their identities as clinicians tasked with providing medical and pastoral care to the patient sitting in front of them, not to their role as bureaucratic administrators focused on policing access to social grants.

The Results of this small study should be treated with some caution, as they are not necessarily representative of wider trends in assessment practices across the country. This is partly because this sample of doctors were all neurodevelopmental paediatricians based at specialised hospitals in one (relatively well-resourced) province and were therefore likely to have had similar training and contextual influences. Other CDG-assessing doctors may have different approaches or beliefs, particularly because while the workloads of the doctors interviewed for this study were very high, they also have better access to human and other health resources when compared to rural or primary care facilities (Burger & Christian, 2020; Coovadia *et al*., 2009; Mayosi & Benatar, 2014; Rispel, 2016). Even so, it is informative to investigate and unpack such subjective experiences because they demonstrate how the practices of these doctors (and others who conduct assessments across the country) may have a considerable impact on the enactment of policy, as highlighted by Dr D’s own contention that ‘in the real world of my clinic, I just don’t see [policy] driving practice’. By making somewhat autonomous choices, these doctors were creating an instantiation of policy that does not necessarily conform to legislation documents, as similarly reported in local and international literature on ‘street-level’ decision-making and its effects on policy implementation (Hoag & Hull, 2017; Lipsky, 2010; Nothdurfter & Hermans, 2018). Rather than seeing these patterns as a threat to implementation, policy-makers could learn from the pragmatic approaches these decision-makers have adopted under constrained circumstances, and could also gain useful insights into where the main pressure points are in the assessments system.

### Strengths and limitations of the research

The initial design of this study included ethnographic observations but these were prohibited due to COVID-19 lockdowns. Such observations might have allowed for the identification of differences between doctors’ self-presentation and their actual behaviour, whereas this research relied only upon self-reporting in one-on-one interviews. Additionally, individuals who respond to requests for qualitative interviews are often among the most forthcoming in a group, which introduces the potential for bias due to self-selection. Finally, it is possible that a social desirability bias was operating. However, the in-depth, semi-structured approach to interviewing offered opportunities to repeatedly interrogate feedback and while there is always a chance that participants felt the need to represent themselves in a positive light, it is unlikely that the position of the interviewer – a younger, less educated person with no clinical knowledge – was especially influential in this regard. Participating doctors were also honest about bending the rules when they felt they had to, as well as about doubting whether they were protecting state resources sufficiently. Their willingness to participate and their candidness suggest that doctors felt comfortable enough to share their real feelings openly. An additional limitation was that due to constraints on in-person contact and research in the public health sector, it was only possible to access doctors working as neurodevelopmental paediatricians, which resulted in a limited sample. We do not suggest that this is necessarily reflective of a national profile of CDG-assessors, nor even of the whole province, as considerable differences may be introduced by the workplace context of the doctor, as well as their relationships with SASSA. However, since there is no other research with CDG-assessing doctors that is currently available, we sought to publish these findings as a first step toward improving this evidence base and importantly, toward the call for including assessors’ perspectives in planning for the implementation of disability-related grants. One of the strengths of this research is that it makes an initial contribution to an as yet unresearched area. Further, only a short time has elapsed since data collection, so this paper offers up-to-date evidence. We recommend that additional research be done across the country, especially in rural settings and with assessors who are based in primary care or non-specialist hospital settings, to complement this study and deepen our understanding of how these practices and beliefs might differ in other settings. This is likely to be particularly important in provinces with high beneficiary numbers, such as KwaZulu-Natal and the Eastern Cape (Hall, 2022), provinces which are also home to large rural populations with low access to specialist care. Rates of approval for the CDG has increased most in the Western Cape in the last decade but the absolute numbers of beneficiaries is significantly higher in these other provinces.

## Conclusion

In this study, doctors expressed their discomfort with their positioning at the interface between an unwieldy bureaucratic system and huge questions about redistributive justice and what the country can afford. Although they may have conducted disability-related grants assessments throughout their careers, these already overloaded doctors did not volunteer for this role and nor was it one of their goals when they chose to train in paediatric medicine. They have been inserted into a large system that is focused on grants administration, without having any specific interest (or training) in social security or social assistance. This is partly a reflection of the potency of doctors’ perceived power, particularly in the process of validating claims on public and private benefits, also the case in other parts of the world. As a society, we have given doctors the power to make these decisions but have not ensured that they have the necessary skills or insights to deliver upon them. These doctors *do* have significant actual and symbolic power, but the impact of this power may be significantly diluted by broader socioeconomic forces, inequities and the medical intractability of the impact of some forms of disability (Watermeyer, 2013). Our participants felt especially disempowered by the structural conditions surrounding their patients, which limited the impact their medical knowledge could have. On top of this, their voices were and are not formally included in conversations about policy and implementation, limiting their capacity to feedback and influence policy from the bottom up. We have also demonstrated in this paper that the crucial perspectives of doctors who conduct assessments for the CDG are not currently being fed back to those who design and adjust policy, and that the ‘trickle down’ of updates to policy are not being clearly or systematically communicated to those who are expected to implement this programme.

Doctors’ narratives are the most direct representations of how assessments for the CDG happen in practice rather than in theory, but these doctors are not in regular contact with SASSA. It is critical that their understanding of factors including severity thresholds (qualification for award of the CDG) and eligibility guidelines, as well as their perspectives about the appropriate way to use the CDG, are captured and understood. In the most substantial prior attempt at revising disability-related grants assessments (due to lack of access or rapidly increasing access), the process of harmonising the tools used for grants assessment with the country’s commitments to disabled people faltered, apparently because of resource limitations and clashing priorities at SASSA and the Department of Health. These previous revision attempts hold valuable lessons that could and should be drawn upon to inspire needed improvements in the current system (Schneider *et al*., 2011b). Far more important than the tools used for assessment, perhaps, is proper collaboration and mutual respect between collaborating departments and government agencies, as well as the ensuing process of informing, reorienting and retraining. As expressed by doctors and SASSA officials alike, SASSA cannot be solely responsible for setting thresholds for inclusion for disability-related grants because they do not hold the relevant expertise. At the same time, however, it is unfair to shift this responsibility onto doctors who are already carrying a huge workload. As Kelly argues, this is also unlikely to be successful because attempting to homogenise applying individuals to a narrow set of categories (e.g. mild, moderate and severe) will be resisted by doctors, who are aware that disability cannot be reduced to these classifications. They may then ‘side-step protocols’ and favour their own professional and experiential knowledge over ‘top-down control’ in the form of overly restrictive guidelines (Kelly, 2016a). Instead, a set of practical and pragmatic guidelines should be co-created, with doctors and SASSA collaborating more closely to find an agreeable approach that takes both perspectives into account. These guidelines must acknowledge the reality of scarcity in all public sector institutions (especially the health system) and the importance of careful relationship management, open communications and a shared understanding of purpose and eligibility in the delivery of this important intervention.

## Supplementary Material

Extended data

## Data Availability

Underlying data The data from this research are not publicly available due to ethical restrictions regarding anonymity and confidentiality. Transcripts and audio data contain identifying information that would compromise the privacy of our research participants. Due to the specificity of these doctors’ roles and the limited number of specialised facilities locally, it would be impossible to completely anonymise the data. Readers may be able to identify hospitals or specific doctors who participated, comprising their confidentiality. Additionally, these data were generated by a specific interaction between the first author and the study participants and was affected by our respective positionality, meaning the data cannot simply be reanalysed by other researchers in the way that survey data might. The data that support the findings of this study are available from the corresponding author and further analysis or collaboration can be requested, in discussion with the authors.

## *1REVISED* Amendments from Version 1

We thank the two reviewers for their valuable input. The following key changes have been made to the updated version of this manuscript:

1. Clarifying text has been added to the Abstract.
2. In response to reviewer comments, the third paragraph in the Introduction notes and briefly expands upon the idea that the problems around disability assessment for publicly funded benefits are not uniquely South African and the same section notes that all of the doctors in our sample were ‘treating doctors’, which is not the norm for disability-related grants across the country.
3. In response to reviewer comments, some details about the CDG have been added to the Background section, while others have been removed as they are less important. The Background section has also been reorganised for more effective communication. We have also expanded upon what we mean by ‘worthy’ and ‘deserving’, per one reviewer’s comments.
4. Additional details regarding recruitment during COVID-19 have been added to the Methods and Strengths and Limitations sections.
5. The now-second paragraph of the Discussion was moved up from lower in the Discussion, for improved flow, i.e. from summary to analysis, rather than back and forth between them. A caution regarding the limitations of these Results has also been included as a new, final paragraph in the Discussion, in response to a request from one of the reviewers.
6. A paragraph that was originally part of the Discussion has been moved to the Conclusion, which has also been abbreviated and restructured for clarity and improved focus.
7. Finally, minor typographical and grammatical changes have been made throughout for clarity and to remove repetition. These edits have not substantively changed the meaning of the sentences in question.

**Any further responses from the reviewers can be found at the end of the article**
