# Supplementary material for: ‘Five minutes earlier, you were giving hope’: Reflections from interviews with doctors conducting assessments for South Africa’s childhood disability Care Dependency Grant
Source: Wellcome Open Res. Author manuscript; Available in PMC 2023 Feb 10. (PMC7614167)
Supplement: Extended data [file EMS164594-supplement-Extended_data.docx]

**Semi-structured interview guide:**

**Doctors involved in conducting medical assessments of childhood disability for social assistance**

***Introduction/consent refresher:***

Thanks again for agreeing to talk with me today. Did you have any questions about the informed consent form I sent you? Are you comfortable with signing it? Is it ok for me to start audio recording now?

To remind you, I don’t work for the government or the media. I won’t make money from this project. I am a researcher from Stellenbosch University. I am doing research for my doctoral degree about how families use and access the care dependency grant (CDG), and what other kinds of support are available to them. I would like to understand more about your work with children with disabilities and their families. I might take some notes while we are talking, and I will use a list to remind myself of the questions I want to ask.

I plan to share this information with other researchers who are interested in social security for disabled children and their families in South Africa. I also hope to tell SASSA what works well and if the system could be improved. I won’t share your name with anybody. At the end of the interview, I will ask you if you would like to choose a fake name for yourself.

If you have any questions during the interview, please stop me and ask. If there is any question you don’t want to answer, please say so – and you don’t need to explain why. How much time do you have to talk today? Ok, let’s start!

***---***

***Demographics, background training, and work experience:***

I’d like to ask you some background questions about you first, if you don’t mind. You don’t have to answer any questions that make you uncomfortable.

Please tell me how old you are, and what race and gender you are? Please use words you are comfortable with.

What training did you do after finishing school?

What is your current job title? How long have you been doing this job?

Can you tell me a little bit about what your day to day work looks like?

***Issues around assessment for and opinions of the CDG:***

My research is focused on the care dependency grant. As you know, the CDG is for caregivers of children with disabilities with specific care needs.

Have you ever been involved in conducting medical assessments for the CDG?

If so, please could you tell me a bit about that process? (*prompt: What forms are used? What medical documentation is used as “evidence”? Where does the assessment happen? What steps are taken from when the client arrives to when the assessment finishes?*)

Which parts of the assessment process work well?

And what challenges do you face?

What do you think the main purpose of the CDG is?

What are the eligibility rules for the CDG? (*prompt: In other words, when do you recommend a CDG is awarded?* *Can you recall times when you did not approve the grant, and why not?*)

What are the factors you think about when you decide who should or should not get the CDG?

What is the most impactful factor, when you are considering if a caregiver needs the CDG?

Can you tell me a bit about how you feel about the fact that the grant is provided from when it is approved until the child is 18? (*prompt: Is this something you consider when you are recommending or not recommending a grant?*)

Do you also conduct assessments for the adult disability grant?

If so, is there a difference in the way you think about these assessments? Can you comment on what you think about when assessing adults rather than children for disability social assistance?

How do you think caregivers who receive the CDG spend the grant money?

How do you feel about this?

Do you think the CDG helps families to manage the challenges related to their child’s disability?

If so, how so?

If not, why not?

Do you see any positive impacts of the CDG?

If so, please tell me a bit more?

Do you see any negative impacts of the CDG?

If so, please tell me a bit more?

***SASSA, the CDG assessment/application process, and government social support:***

Were you contracted to SASSA, or do you work in the public sector?

Have you conducted assessments at clinics only, or have you also done them at hospitals?

When assessing a caregivers' eligibility, do you feel like you have enough guidance from SASSA?

What kinds of guidance do you receive?

Did you ever receive specific training for CDG or other disability grant assessments?

Can you tell me a bit about the training?

Do you ever get updated or refresher training?

Have you ever been in contact with SASSA or the Department of Social Development about any aspect of the CDG?

If so, what was that like?

Do you ever get a chance to give feedback to SASSA?

If SASSA asked you for your ideas to improve the assessment process or the rules about eligibility, what would you tell them?

***Understandings of disability:***

Finally, I’d like to talk a little bit more about disability.

Can you tell me what your definition of “disability” is? Say you have a new colleague who has never worked with disability before and they ask you: “what do you mean by a disability?” Can you tell me how you would answer them? Take your time.

Besides the grant, do you think the government should do anything else to help caregivers of children with disabilities?

What kinds of help would be most useful/what do you think families need most?

Why?

---

Ok, we are finished! Do you have any questions for me?

Would you like to choose a pseudonym for yourself? Otherwise, I will make one up.

I want to remind you again that although you will be completely anonymous, it is possible that somebody will read this and recognise your job position. If you would like, I will make sure to send you a draft of anything that uses information you have provided, and you can let me know if you would like me to remove anything.

Thanks again for agreeing to talk to me. Remember, my contact details are on the consent form I sent you – please contact me any time if you have any questions or concerns later.
