# Supplementary material for: ‘Five minutes earlier, you were giving hope’: Reflections from interviews with doctors conducting assessments for South Africa’s childhood disability Care Dependency Grant
Source: Wellcome Open Res. 2022 Nov 25;7:263. Originally published 2022 Oct 14. [Version 2] doi: 10.12688/wellcomeopenres.18424.2 (PMC7614167; doi:10.12688/wellcomeopenres.18424.2)
Supplement: Peer review history

# Peer review: Reviewer response for version 2

**DOI:** 10.21956/wellcomeopenres.20602.r53444

**Published:** 2023-01-11

**Authors:** Gabrielle Kelly

**Affiliations:** 1 Samson Institute for Ageing Research, Cape Town, South Africa

I am happy with the revisions made.

Is the work clearly and accurately presented and does it cite the current literature?

Yes

If applicable, is the statistical analysis and its interpretation appropriate?

Not applicable

Are all the source data underlying the results available to ensure full reproducibility?

No source data required

Is the study design appropriate and is the work technically sound?

Yes

Are the conclusions drawn adequately supported by the results?

Yes

Are sufficient details of methods and analysis provided to allow replication by others?

Yes

Reviewer Expertise:

Social protection, disability grants, care for older persons

I confirm that I have read this submission and believe that I have an appropriate level of expertise to confirm that it is of an acceptable scientific standard.

# Peer review: Reviewer response for version 2

**DOI:** 10.21956/wellcomeopenres.20602.r53443

**Published:** 2022-12-12

**Authors:** Marie Schnitzler

**Affiliations:** 1 Institute of Research in Social Sciences, University of Liège, Liège, Belgium

The authors have diligently answered my previous comments. I thank them for this very interesting paper that I enjoyed reading.

Is the work clearly and accurately presented and does it cite the current literature?

Yes

If applicable, is the statistical analysis and its interpretation appropriate?

Not applicable

Are all the source data underlying the results available to ensure full reproducibility?

Yes

Is the study design appropriate and is the work technically sound?

Yes

Are the conclusions drawn adequately supported by the results?

Yes

Are sufficient details of methods and analysis provided to allow replication by others?

Yes

Reviewer Expertise:

Anthropology, social sciences, disability studies

I confirm that I have read this submission and believe that I have an appropriate level of expertise to confirm that it is of an acceptable scientific standard.

# Peer review: Reviewer response for version 1

**DOI:** 10.21956/wellcomeopenres.20431.r52864

**Published:** 2022-11-02

**Authors:** Marie Schnitzler

**Affiliations:** 1 Institute of Research in Social Sciences, University of Liège, Liège, Belgium

This paper offers an interesting insight into the ambiguity of disability-related social assistance cash transfers in South Africa. More specifically, it shows the intertwining of medical and functional approaches of disability, together with more circumstantial element, when doctors take administrative but very impactful decisions. As such, it probes into the technical dimension of the assessment as well as the conflict of values doctors have to resolve when advising for the delivery of the care dependency grant.

 A few modifications or further details might help improve the paper:

1. Providing some information on the number of CDG could help the reader better understand some arguments of the paper such as the actual economic importance of the grant, or the difference in terms of recipients with other grants such as the CSG or the DG grant. This information is easily retrieved from SASSA reports.
2. Some clarity on what the authors mean by “worthiness” and “deservingness” might be needed to avoid any confusion. Indeed, the authors estimate that recipients’ “worthiness” is not much discussed by doctors (which differs from the DG assessment, see p.9), but these doctors still comment on the emotional reactions steered by patients’ condition and circumstances, both negative and positive (p.5). Is taking into account these elements not partly estimating the deservingness of some in front of other? Or is the discussion on worthiness more related to identifying fraudulent applicants?
3. When limitations are discussed, the question of selection is never mentioned. Indeed, the fact that doctors “eagerly agreed to be interviewed” (p.10) interrogate the participants’ profile. Can we assume that all paediatric doctors are “softies” or are the data putting this profile to the front because doctors who participate in the research were the ones the most concerned with providing care for their patients and just assessments?  A few information on the number of requests for interview and some cautions around the results could help bring a more nuanced tone. This however does not mean that the results are invalid, only that the method of recruitment might have favour certain type of participants’ profiles.
4. Somehow linked with the previous comments, when the authors underline the need for further studies in rural areas, adding other types of medical personnel providing CDG assessment beyond neurodevelopmental paediatricians could be useful.
5. In the conclusion, the need to review the actual system is not really contextualised. What is shown in the paper is a system that ensures flexibility, although it puts a lot of responsibilities and doubts on doctors. But why should this system be revised is not completely clear to me. Is this in link with SASSA desire to standardise the access to the grant and thus is SASSA already planning to modify the system of assessment? Or is there a real call from doctors to change the way they provide assessment, not only during interviews but publicly voiced?

Is the work clearly and accurately presented and does it cite the current literature?

Yes

If applicable, is the statistical analysis and its interpretation appropriate?

Not applicable

Are all the source data underlying the results available to ensure full reproducibility?

Yes

Is the study design appropriate and is the work technically sound?

Yes

Are the conclusions drawn adequately supported by the results?

Yes

Are sufficient details of methods and analysis provided to allow replication by others?

Yes

Reviewer Expertise:

Anthropology, social sciences, disability studies

I confirm that I have read this submission and believe that I have an appropriate level of expertise to confirm that it is of an acceptable scientific standard, however I have significant reservations, as outlined above.

# Peer review: Reviewer response for version 1

**DOI:** 10.21956/wellcomeopenres.20431.r52863

**Published:** 2022-10-31

**Authors:** Gabrielle Kelly

**Affiliations:** 1 Samson Institute for Ageing Research, Cape Town, South Africa

Thanks for the opportunity to read this well crafted article. I feel that you have addressed many logistical, bureaucratic, medical, social and interpersonal issues that make it so difficult for doctors to conduct their assessments "objectively". The article could be strengthened in the following ways:

1. Making it explicit in the methodology that your sample are all treating doctors (you indicate upfront that they are all at specialist hospitals but not that they are treating patients). This is not the norm in SA or elsewhere. For those readers that don't understand the MoU between DSD and SASSA it might be helpful just to make this clear upfront as there are obviously differences between their approach and those that those hired specifically as disability assessors will have, which you have touched on in your analysis but I think should come earlier. You should probably also highlight this as a limitation as you only have the perspectives of a very *specific *group of specialists with relationships with their patients who are being seen at a higher level of the health system. This is not to say that the perspectives of these doctors are not tremendously useful or important, but I do think you need to position them upfront - I know you are not trying to generalise, but it is still important.
2. I think you need to reflect on the implications of doctors work on policy implementation. You have noted that they should be involved in implementation planning, which I fully agree with. But what might be the consequences of their current practices? Street-level bureaucracy theory posits that bureaucratic practice "creates" policy - even if you don't employ this theory directly, it would be useful to reflect on what doctors' behaviour and gatekeeping role means for access to the system. It's a big question and you may not have space to do this in detail, but I think at least pointing to some of the practical implications of their practice and some of the established thinking and literature in the area of policy implementation and how your research extends or contributes to this would be very helpful.
3. I think you also need to highlight that these struggles are by no means a local problem. Disability assessment is notoriously difficult and doctors are notoriously bad at doing it all over the world. Socio-economic issues aside, the medical model is very limiting and doctors are not trained to assess function. The South African case is so interesting because it takes place in a context where there are much higher levels of poverty than in many other countries with disability programmes and it accentuates the challenges. While disability benefits for children are less politicised than for adults, some of this research has focused on disability benefits for children in the American context. I think that linking it to broader international challenges and political debates might bring your article to a broader audience. 

Is the work clearly and accurately presented and does it cite the current literature?

Yes

If applicable, is the statistical analysis and its interpretation appropriate?

Not applicable

Are all the source data underlying the results available to ensure full reproducibility?

No source data required

Is the study design appropriate and is the work technically sound?

Yes

Are the conclusions drawn adequately supported by the results?

Yes

Are sufficient details of methods and analysis provided to allow replication by others?

Yes

Reviewer Expertise:

Social protection, disability grants, care for older persons

I confirm that I have read this submission and believe that I have an appropriate level of expertise to confirm that it is of an acceptable scientific standard, however I have significant reservations, as outlined above.
